# Relation of nailfold capillaries and autoantibodies to mortality in patients with Raynaud’s phenomenon: a retrospective analysis

**DOI:** 10.1007/s00296-026-06261-7

**Published:** 2026-08-04

**Authors:** Markus Müller, Michael E. Gschwandtner, Hans Kiener, Thomas Perkmann, Sonja Zehetmayer, Sophie Brunner-Ziegler, Sabine Steiner, Oliver Schlager

**Affiliations:** 1 Division of Angiology, Department of Medicine II, Medical University of Vienna, Vienna, Austria; 2 Division of Rheumatology, Department of Medicine III, Medical University of Vienna, Vienna, Austria; 3 Department of Laboratory Medicine, Medical University of Vienna, Vienna, Austria; 4 Center for Medical Data Science, Medical University of Vienna, Vienna, Austria

**Keywords:** Raynaud phenomenon, Mortality, Nailfold capillaroscopy, Microvascular dysfunction, Small-vessel disease

## Abstract

The presence of abnormal nailfold capillaries and antinuclear antibodies (ANA) is associated with increased all-cause mortality in patients presenting with incipient Raynaud’s phenomenon (RP). We conducted a retrospective analysis aiming to assess the association between abnormal nailfold capillaries, ANA, and mortality over a 20-year follow-up period. In 2958 patients with incipient RP without previously known connective tissue disease, nailfold capillaroscopies and laboratory tests for ANA and ANA-subsets were obtained. Of these, long-term follow-up data were available for 2922 patients. Over a median follow-up period of 23 (IQR 20.2–25.2) years, 832 patients died, of which 562 were women (25% of female patients) and 270 were men (41% of male patients). Compared to a demographically matched reference population, mortality was increased in female and male patients with RP (log-rank test < 0.001). In female patients with RP, the presence of ANA, anti-Scl-70 antibodies, and a grouping variable consisting of giant capillaries, reduced capillary density, and avascular fields was associated with an increase in all-cause mortality. The highest mortality was observed in patients presenting both abnormal nailfold capillaries and ANA. In the long-term, an excess mortality persists in patients with RP. Our study demonstrates prognostic associations between abnormal nailfold capillaries as well as ANA and mortality.

**Supplementary Information:**

The online version contains supplementary material available at 10.1007/s00296-026-06261-7.

## Key messages

- What is already known on this topic:
  - Patients with incipient Raynaud’s phenomenon, who exhibit abnormal nailfold capillaries and antinuclear antibodies, have a higher mortality than those with neither of the two.
- What this study adds:
  - This study confirms an association between abnormal nailfold capillaries, autoantibodies and mortality in patients with incipient Raynaud’s over a long-term follow-up of twenty years.
- How this study might affect research, practice or policy:
  - Further studies are needed to elucidate the link between a potential immune-mediated microangiopathy and mortality in a subset of patients with incipient Raynaud’s phenomenon.

## Introduction

Raynaud’s phenomenon (RP) is a common vasospastic disorder that manifests as paroxysmal pallor, cyanosis, and occasionally reactive hyperemia of the hands after exposure to cold or emotional stress [1]. Although the exact complaints may vary from individual to individual, abnormal sensitivity to cold, as well as cold-related pallor or cyanosis, are considered hallmarks of the disease. Symptoms of RP may also occur at other sites, such as the toes, ears, or nose, and paresthesia, a throbbing sensation, or outright pain may accompany attacks [2] Whereas primary RP has mostly been considered a benign condition resulting from episodic vasospasm of digital arteries, secondary RP has been linked to systemic autoimmune rheumatic disease, which it may precede by several years, and can be associated with irreversible structural changes in the capillary architecture detectable by nailfold capillary microscopy [3, 4]. The differentiation between the two types is thus the focus of the initial clinical examination in patients presenting with RP, and studies usually performed to this end comprise nailfold capillaroscopy and, in the case of pertinent clinical signs or symptoms compatible with connective tissue disease (CTD), immunological testing for antinuclear antibodies (ANA) and ANA-subsets [5–9]. Previously, we found an increased mortality in patients with incipient RP without previously known CTD over a follow-up period of almost 10 years [10]. Following these findings, we aimed to assess the long-term association among nailfold capillaroscopy, ANA, and mortality in patients presenting with incipient RP without a previously known CTD. To this end, a 20-year follow-up in a cohort of 2922 patients with incipient RP was performed, using data obtained from the respective national registry, the Austrian Statistics of Death (Statistik Austria).

## Methods

### Patients

Patients with RP and without known CTD who presented to the vascular outpatient clinic of the Vienna General Hospital, a tertiary care center between January 1994 and April 2008 were eligible for the present study. The inclusion criterion for study participation was a clinical diagnosis of RP without a previously known CTD, while exclusion criteria included hemodynamically relevant upper-extremity arterial obstructions as well as clinical or anamnestic signs of secondary RP. Potential differential diagnoses such as vibration white finger and carpal tunnel syndrome were excluded based on physical examination and history taking. All study participants were initially referred for oscillographic pulse volume measurements of both arms and digital pulse wave analysis to exclude relevant macroangiopathy. Patients then underwent nailfold capillaroscopy and serological testing for ANA and, at the attending physician’s discretion, ANA subsets. This study was performed in accordance with good clinical practice guidelines and was approved by the ethics committee of the Medical University of Vienna (731/2010).

### Nailfold capillaroscopy

All study participants underwent nailfold capillaroscopy of both hands. Nailfold capillaroscopy is a non-invasive, sensitive, and cost-effective means of assessing the digital microcirculation. Examinations were performed under standardized conditions in a quiet room at 23 °C, following a 20-minute acclimatization period in a sitting position. Manicure was discouraged at least one week before the examination, and participants were asked to remove any nail polish before capillaroscopy. Fingers 2 to 5 of both hands were examined. Capillaroscopic findings were registered as previously described: (1) normal nailfold capillary pattern, (2) reduced capillary density (< 30 hairpin-shaped capillaries per 5 mm), (3) avascular fields (a large capillary-free, dessert-like area), (4) dilated capillaries (a capillary diameter > 20 μm but < 50 μm), (5) giant capillaries (a maximum capillary diameter > 50 μm), (6) hemorrhages (hemosiderin extravasations), (7) tortuous capillaries, (8) ramifications, and (9) capillary edema (capillary blurring or pericapillary halo).^[10]^ A capillary microscopic system (CAM1, KK Research Technology LTD and Leitz, Wetzlar) with a 250-/300-fold magnification was used for all nailfold capillaroscopic examinations. The obtained images were recorded and processed using the CapiScore software (KK technology). All examinations were supervised by one experienced investigator (MEG), and registered images were then independently classified by two investigators (OS, MEG). For study purposes, the findings were denoted as either normal nailfold capillaries (absence of nailfold capillary aberrations) or abnormal nailfold capillaries (presence of at least one capillary aberration). As some of the mentioned capillary aberrations, such as capillary dilations or tortuous capillaries, may also be found in healthy individuals,^11^ or may be the result of nailfold manipulation, as is the case for capillary hemorrhages, we introduced the additional category of pathognomonic nailfold capillary changes. This category denotes capillary aberrations that are not encountered in healthy individuals and that testify to the presence of microvascular damage. The category comprised giant capillaries, a reduced capillary density and avascular fields and in the presence of either of these capillary aberrations study participants were classified as displaying pathognomonic capillary changes, which were included in this retrospective analysis as a derived variable.

### Serological tests

All study participants underwent venipuncture and laboratory testing to determine ANA titers. Indirect immunofluorescence on Hep-2 cells (Kallestad, Bio-Rad Laboratories, Hercules, CA) was performed at the institutional laboratory, and an ANA-titer of ≥ 1:160 was considered positive. Additionally, ANA subsets (anti-Scl-70, anti-CENP-B, anti-SSA (Ro), anti-SSB (La), anti-U1-RNP, anti-SM, anti-Jo-1, and anti-ds-DNA antibodies) were tested by immunologic assays in patients with clinical suspicion of an underlying connective tissue disease, as well as in participants with specific immunofluorescence patterns on Hep-2 Cells. As the availability of respective assays varied over time, no single method for immunologic determination of ANA was used throughout the study period. As previously described, Ouchterlony immunodiffusion (Auto I.D., Immuno Concepts, Sacramento, CA) was used from 1994 to September 2005. Multiplex fluorescent technology (FIDIS Connective 10, BMD, France) was used from September 2005 to January 2007, and the UniCAP Elia System (Phadia, Sweden) was utilized thereafter.

### Morbidity and mortality

Mortality data of study participants were obtained from the respective national registry, the Austrian Statistics of Death (Statistik Austria), as were the causes of death, which were, for study purposes, further classified as malignant neoplasms, ischemic cardiovascular disease, nonischemic heart disease, renal failure and other causes of death (such as pulmonary diseases, infectious diseases, gastrointestinal diseases, liver diseases, immunologic diseases, hematologic diseases, drug/alcohol abuse, and trauma). To account for cardiovascular risk factors, patients’ records were screened for the presence of arterial hypertension, hyperlipidemia, obesity (defined as body mass index ≥ 30), diabetes mellitus, and a history of smoking. Additionally, serum creatinine and corticosteroid medication were also recorded.

### Standard population

To put mortality data for study participants into perspective, an age- and sex-matched standard population was used for comparison. Respective survival data were provided by the Austrian Statistics on Deaths (Statistik Austria) in the form of life tables. The respective life tables contain an individual’s estimated further life expectancy as derived from the reference population (i.e. the Austrian general population) for each year from 1947 to the present. The provided estimates are sorted with respect to sex and age at the respective year, for which the further estimated life expectancy is to be obtained (e.g. 25-year-old male individual in the year of 1994). Based on age, sex, and the year of initial examination of our study participants, a respective standard population can be obtained, and the observed survival of study participants and the standard population can be compared.

### Patient and public involvement statement

Patients and the public were not involved in the design and conduct of this study due to its retrospective nature.

## Statistical analysis

Continuous data are given as median and interquartile range. Discrete data are given as counts (n) and percentages (based on the number of study participants N). Differences between male and female patients were analyzed using Mann-Whitney U tests and χ^2^ tests as appropriate. The median follow-up time was calculated using a reverse Kaplan-Meier model. To compare the mortality of study patients with that of a demographically matched standard population, life tables were obtained from Statistik Austria. Kaplan-Meier analyses and log-rank tests were used to examine differences in survival between the study population and the demographically matched standard population. In order to examine the relation of age, sex, serum creatinine, normal/abnormal nailfold capillaries, as well as distinct autoantibodies to mortality, univariable Cox proportional hazards models were calculated. A grouping variable was included, comprising the presence of giant capillaries and/or reduced capillary density and/or avascular fields. All predictors that showed an association with mortality at an α-level of ≤ 0.1 were then included in a multivariable Cox proportional hazards model. The absolute risks (in this case corresponding to the respective positive predictive values), the relative risks, as well as the negative predictive values of abnormal nailfold capillaries and distinct antibodies with respect to ten-year mortality were calculated. To assess for differences in outcome as a function of sex, all analyses were stratified accordingly. To differentially assess the impact of abnormal nailfold capillaries and the presence of autoantibodies on patients’ mortality, study participants were stratified into four groups, based on capillaroscopic and serologic findings: group 1: normal nailfold capillaries, absent autoantibodies; group 2: abnormal nailfold capillaries, absent autoantibodies; group 3: normal nailfold capillaries, present autoantibodies; and group 4: abnormal nailfold capillaries and present autoantibodies. Differences in survival between these groups were examined using Kaplan-Meier analysis and log-rank tests. All statistical calculations were done using the software package R version 4.5.1 (http://cran.r-project.org/).

## Results

In total, 2958 patients with incipient RP and no prior CTD were eligible for this follow-up study. Of these, long-term follow-up data were available in 2922 patients (median age 46 years; 2268 women, 77.6%). Median age at study inclusion was 44 (IQR 32–57) years in women and 51 (IQR 38–61) years in men. The prevalence of abnormal nailfold capillary patterns, ANA, ANA subsets, and serological findings is shown in Table 1. The median follow-up time was 23 (IQR 20.2–25.2) years.

**Table 1:** Findings of capillaroscopy and serologic analysis in 2922 patients with incipient raynaud phenomenon according to sex

	Females (*N* = 2268)	Males (*N* = 654)	*P* value
Normal capillary pattern, *n*/*N* (%)	554/2268 (24)	165/654 (25)	0.68
Capillary dilations, n/N (%)	1579/2268 (70)	450/654 (69)	0.73
Hemorrhages, n/N (%)	443/2268 (20)	124/654 (19)	0.79
Reduced capillary density, n/N (%)	425/2268 (19)	117/654 (18)	0.66
Tortuous capillaries, n/N (%)	333/2268 (15)	103/654 (16)	0.54
Giant capillaries, n/N (%)	282/2268 (12)	60/654 (9.2)	0.03
Ramifications, n/N (%)	217/2268 (9.6)	45/654 (6.9)	0.04
Edema, n/N (%)	62/2268 (2.7)	20/654 (3.1)	0.76
Avascular fields, n/N (%)	41/2268 (1.8)	10/654 (1.5)	0.76
ANA, n/N (%)	686/2268 (30)	91/654 (14)	< 0.001
Anti-Scl-70, n/N (%)	42/1779 (2.4)	15/513 (2.9)	0.58
Anti-SSa(Ro), n/N (%)	115/1906 (6)	7/543 (1.3)	< 0.001
Anti-SSB(La), n/N (%)	115/1930 (3.2)	3/545 (0.6)	0.001
Anti-U1-RNP, n/N (%)	60/1906 (3.1)	13/541 (2.4)	0.47
Anti-Sm, n/N (%)	10/1902 (0.5)	0	0.19
Anti-Jo1, n/N (%)	4/1720 (0.2)	0	0.64
Anti-ds-DNA, n/N (%)	59/1551 (3.8)	2/416 (0.5)	0.001
Creatinine (mg/dl)	0.87 (0.79–0.96)	1.02 (0.92–1.14)	< 0.001
Erythrocytes (10^3^/µl)	4.5 (4.2–4.7)	4.8 (4.5–5.1)	< 0.001
Hemoglobin (g/dL)	13.3 (12.4–14)	14.7 (13.7–15.5)	< 0.001
Platelets (*10^3^/µl)	258 (216–306)	233 (198–294)	< 0.001
Fibrinogen (mg/dl)	328 (278–392)	334 (271–408)	0.63
C-reactive protein (mg/dL)	0.5 (0.5–0.5)	0.5 (0.5–0.54)	0.11

Data are given as counts (n) and percentages (%) of female/male patients. Percentages refer to the number of tested patients (n). Age and laboratory values are stated as median and inter-quartile range

*ANA* antinuclear antibodies

### Mortality of patients with Raynaud’s phenomenon

**Fig. 1:**
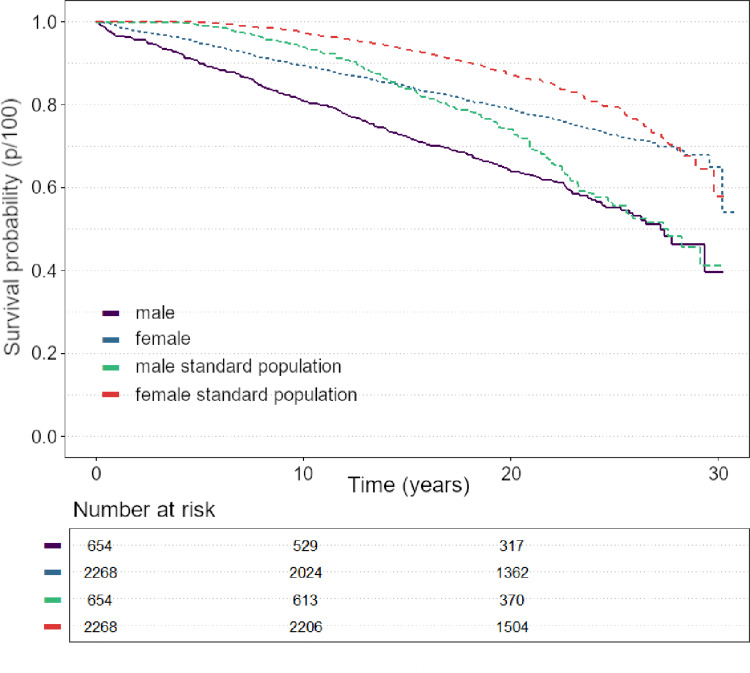
Kaplan-Meier curves depicting survival probabilities and 95% confidence intervals in female and male patients with incipient Raynaud phenomenon as well as in a demographically matched reference population: female patients with Raynaud phenomenon versus female reference population log-rank test P < 0.0001; male patients with Raynaud phenomenon versus male reference population log-rank test P = 0.01. Kaplan-Meier plots depict crude survival curves, do not account for covariables and should be interpreted in the context of the Cox proportional hazards models provided in the manuscript

Over the follow-up period, 832 deaths occurred. Of these, 562 were women (25% of included women) and 270 were men (41% of included men). The median age at death of study participants was 73 (IQR 63–82) years for men and women combined, 73 (IQR 64–83) for women, and 74 (IQR 63–82) for men. Compared to a demographically matched reference population, mortality was increased in patients with RP (log-rank test < 0.001; Fig. 1).

### Nailfold capillaries, autoantibodies, and long-term survival

**Fig. 2:**
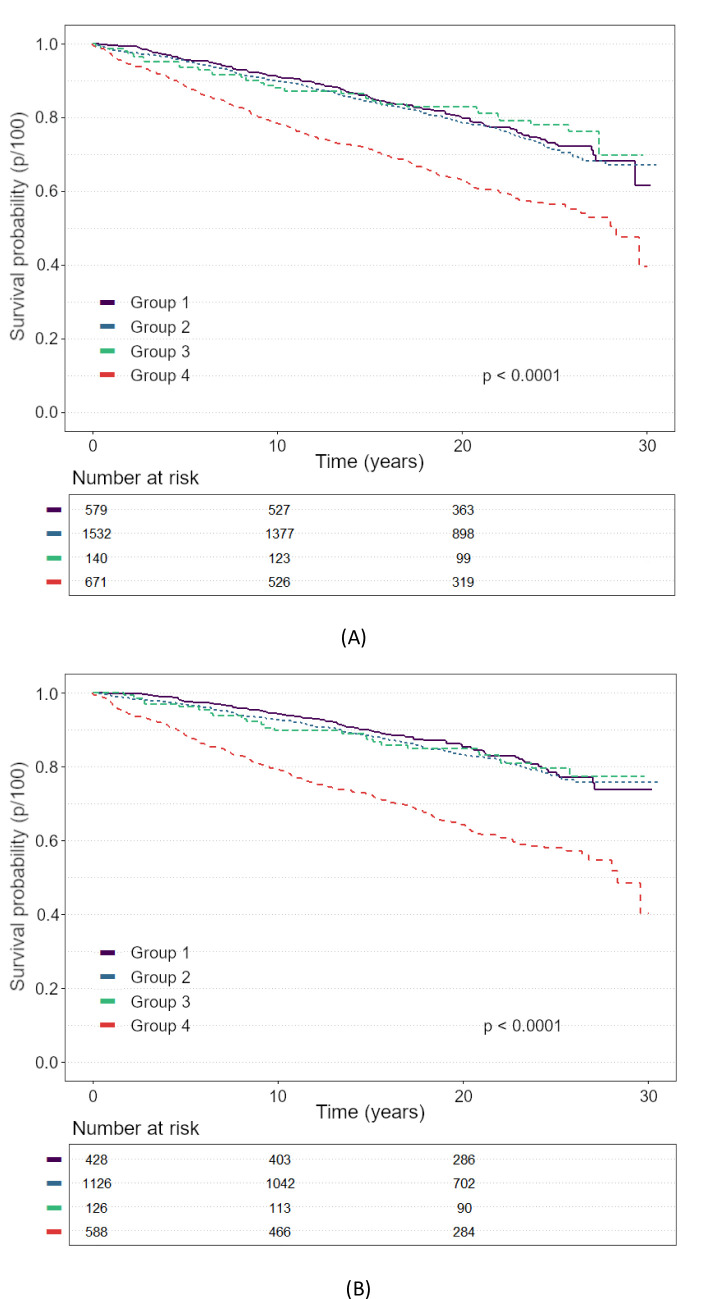

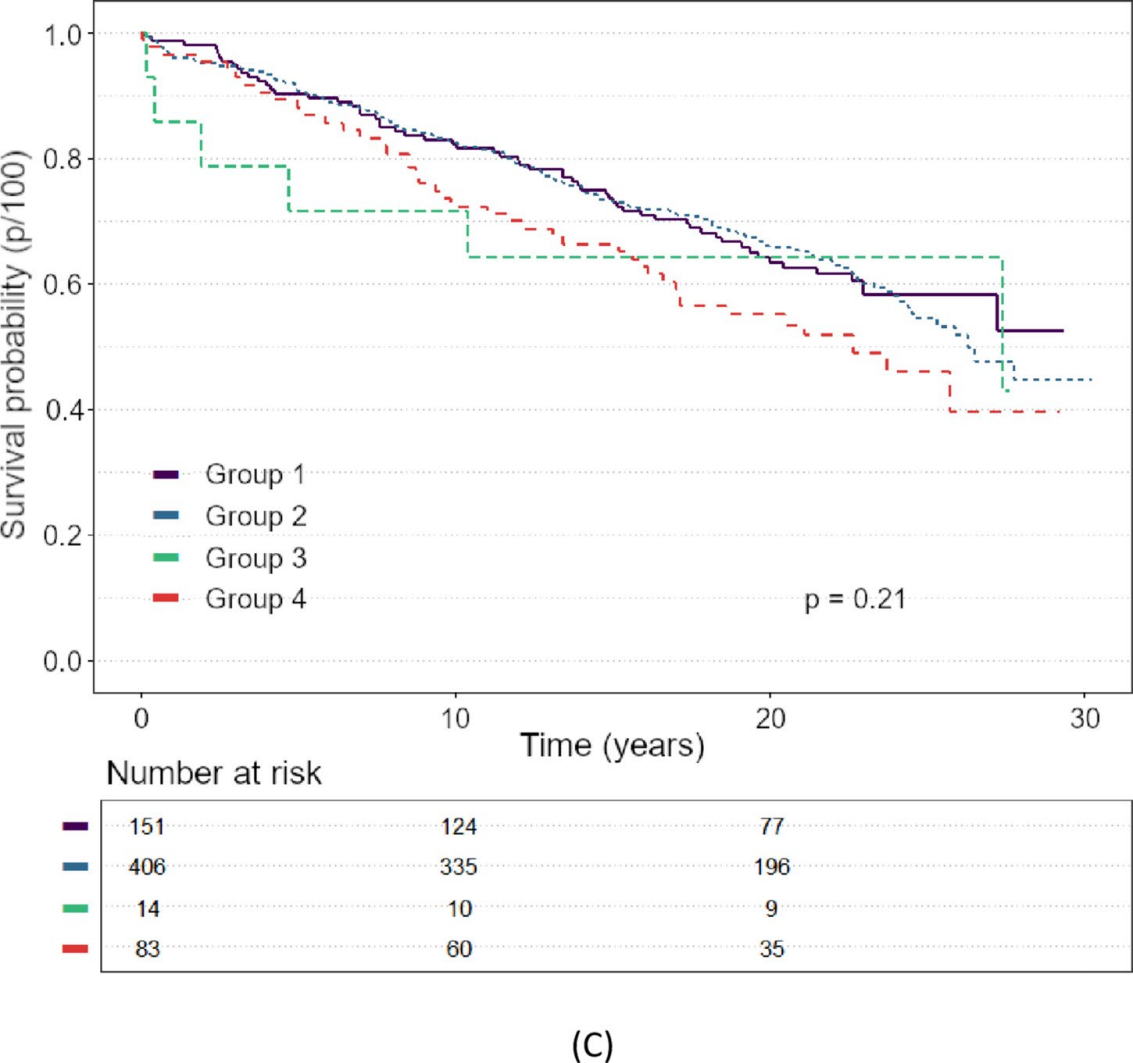
Kaplan-Meier curves depicting survival probabilities and 95% confidence intervals of **A** all study participants with incipient Raynaud phenomenon divided into 4 groups (group 1: normal nailfold capillaries, negative autoantibody tests; group 2: abnormal nailfold capillaries, negative autoantibody tests; group 3: normal nailfold capillaries, positive autoantibody tests; group 4: abnormal nailfold capillaries, positive autoantibody tests); and **B** female patients only stratified according to group and (**C**) male patients only stratified according to group. Kaplan-Meier plots depict crude survival curves, do not account for covariables and should be interpreted in the context of the Cox proportional hazards models provided in the manuscript

The associations between abnormal nailfold capillaries, positive autoantibodies, and mortality are presented in Table 2. Absolute risks, relative risks, and negative predictive values of abnormal nailfold capillaries and serological parameters are shown in Table 3. When patients were stratified by abnormal nailfold capillaries and autoantibodies, the highest mortality was observed in participants who displayed both (log-rank test < 0.001; Fig. 2A and B), although this was not true in the subgroup of male patients (Fig. 2C). In female, but not in male patients, the presence of pathognomonic nailfold capillaries (HR 1.59; 95% CI 1.23–2.04, *p* < 0.001) as well as ANA (HR 1.44; 95% CI 1.13–1.83, *p* < 0.01) and anti-Scl-70-antibodies (HR 2.2; 95% CI 1.44–3.36, *p* < 0.001) were associated with mortality (Table 4).

**Table 2:** Associations of abnormal nailfold capillaries and immunologic markers with mortality in 2922 patients with incipient Raynaud phenomenon (according to sex)

	Hazard ratio	Females (*N* = 2268)			Males (*N* = 654)		*P* value	
		Confidence interval	*P* value	Hazard ratio	Confidence interval			
Abnormal nailfold capillaries	1.51	1.23–1.87	< 0.001	1.08	0.82–1.42		0.60	
ANA	2.11	1.79–2.50	< 0.001	1.30	0.94–1.79		0.12	
Anti-Scl-70	4.36	2.95–6.44	< 0.001	1.55	0.76–3.14		0.23	
Anti-SSa(Ro)	1.54	1.11–2.13	0.01	1.10	0.35–3.43		0.87	
Anti-SSb(La)	1.59	1.02–2.49	0.04	N/A	N/A		N/A	
Anti-U1-RNP	1.06	0.63–1.77	0.83	0.47	0.15–1.48		0.20	
Anti-Sm	0.80	0.20–3.20	0.75	N/A	N/A		N/A	
Anti-Jo-1	1.28	0.18–9.09	0.81	N/A	N/A		N/A	
Anti-ds-DNA	0.79	0.45–1.36	0.39	N/A	N/A		N/A	

*ANA* antinuclear antibodies, *N/A* not applicable due to a low overall prevalence and/or a low number of events in the respective subgroup

**Table 3:** Absolute risks, relative risks, and NPVs of abnormal nailfold capillaries and immunologic markers for 10-year mortality in 2958 patients with incipient raynaud phenomenon (according to sex)

	Females (*N* = 2268)					Males (*N* = 654)			
	Absolute risk (%)	Relative risk (%)	NPV (%)	Absolute risk (%)		Relative risk (%)		NPV (%)	
Abnormal nailfold capillaries	12 (11–14)	1.75	93	19 (16–23)		1.02		81	
Pathognomonic capillary aberrations	20 (16–23)	2.45	92	25 (19–33)		1.46		83	
ANA	19 (16–22)	2.72	93	29 (20–39)		1.62		82	
Anti-Scl-70	38 (24–54)	3.78	90	33 (13–61)		1.75		81	
Anti-SSA(Ro)	15 (9–23)	1.39	89	0 (0–44)		N/A		81	
Anti-SSB(La)	20 (11–33)	1.89	89	0 (0–69)		N/A		81	
Anti-U1-RNP	14 (6–26)	1.25	89	8 (0–38)		0.41		81	
Anti-Sm	10 (1–46)	0.92	89	N/A		N/A		N/A	
Anti-Jo-1	25 (1–78)	2.37	89	N/A		N/A		N/A	
Anti-ds-DNA	7 (2–17)	0.57	88	0 (0–80)		N/A		80	

*ANA* antinuclear antibodies, *N/A* not applicable due to a low overall prevalence and/or a low number of events in the respective subgroup; *NPV* negative predictive value

**Table 4:** Multivariable associations of patients’ age, abnormal nailfold capillaries, ANA-titers as well as ANA subsets with mortality in 2922 patients with incipient raynaud phenomenon (according to sex)

	Females (*N* = 2268)				Males (*N* = 654)		
	Hazard ratio	Confidence interval	*P* value	Hazard ratio	Confidence interval		*P* value
Abnormal nailfold capillaries	1.13	0.84–1.52	0.44	1.10	0.73–1.64	0.66	
Pathognomonic capillary aberrations	1.54	1.21–1.98	< 0.001	1.46	0.99–2.15	0.06	
ANA	1.46	1.15–1.84	< 0.01	0.94	0.59–1.49	0.79	
Anti-Scl-70	2.33	1.54–3.53	< 0.001	2.45	1.10–5.48	0.03	
SSa(Ro)-antibodies	1.11	0.69–1.78	0.67	3.76	1.18–12.00	0.03	
SSb(La)-antibodies	1.04	0.54–2.02	0.91	N/A	N/A	N/A	
Age	1.07	1.06–1.08	< 0.0001	1.09	1.07–1.10	< 0.0001	
Creatinine	1.15	1.03–1.28	0.01	1.29	1.10–1.50	< 0.01	

*ANA* antinuclear antibodies

## Discussion

This long-term follow-up in patients with incipient RP confirms an association between abnormal nailfold capillaries, autoantibodies, and mortality.

Based on the Kaplan-Meier curves, an excess mortality can be observed in patients with incipient RP for almost three decades after first presentation at our outpatient clinic, before differences in survival between patients with incipient RP and controls seem to disappear. We interpret this finding as a consequence of different mortality rates across distinct subgroups within the study population. When patients with incipient RP were stratified by abnormal nailfold capillaries and antibody profile, the highest mortality was observed in individuals with both capillaroscopic evidence of microangiopathy and positive autoantibodies. In contrast, mortality among participants who displayed neither abnormal nailfold capillaries nor serologic abnormalities did not differ from that of the reference population. The Kaplan-Meier curve of patients with incipient RP can thus be viewed as a composite of these subgroups, accounting for the alignment of the respective Kaplan-Meier curves with those of controls in later decades of life. In fact, excess mortality persists throughout the observed period in the subgroup of patients with both nailfold capillary aberrations and autoantibodies (Fig. 2A and B).

Nailfold capillary aberrations per se were not associated with mortality in patients with incipient RP in the Cox proportional hazards models. This can be explained by the fact that capillary changes, such as dilated or tortuous capillaries, as well as hemorrhages, although potentially present in microangiopathy, are nonspecific and can also be found in healthy individuals [11]. Therefore, we included the category of ‘pathognomonic capillary aberrations’ in our analysis, i.e., capillary changes that have been more firmly associated with microangiopathy. Both capillary density and the apical diameter of nailfold capillaries are altered in Scleroderma-associated microangiopathy, and the presence of a reduced capillary density is considered in the microangiopathy evolution score [12]. A reduced capillary density may however also be encountered in conditions as diverse as chronic kidney disease, heart failure, diabetes and essential hypertension [13–15]. The presence of a single giant capillary is considered pathologic and a harbinger of microangiopathy [11]. Pathognomonic capillary aberrations detected at initial examination were associated with increased mortality in patients with incipient RP and without previously known connective tissue disease, as assessed in a multivariable Cox proportional hazards model.

In this context, it should be noted that, according to the American college of rheumatology/European league against rheumatism 2013 diagnostic criteria, the presence of nailfold capillary abnormalities and scleroderma-specific autoantibodies in patients with RP does not suffice to establish the diagnosis of systemic sclerosis in the absence of pertinent clinical findings [16]. Patients with respective findings may however be at increased risk of developing systemic sclerosis in the future and the concept of VEDOSS (very early diagnosis of systemic sclerosis) has been introduced to account for this fact. However, not all patients with VEDOSS will ultimately converse to systemic sclerosis and the term is variably defined in the literature [17]. Moreover, interpretability of the study by Bellando-Randone et al., demonstrating substantial conversion rates from VEDOSS to systemic sclerosis, is limited by a highly-selected patient population recruited at specialized scleroderma trial and research group centers and high rates of censoring, rendering Kaplan-Meier estimates of relative conversion rates less robust [18]. Although a promising concept, the authors feel that additional data would be desirable before applying this diagnosis indiscriminately to RP patients with either nailfold capillary abnormalities or scleroderma-specific autoantibodies.

The presence of both abnormal nailfold capillaries and positive autoantibodies could serve as a surrogate marker of an immunologically driven microangiopathy in a subset of study participants. This microangiopathy may ultimately not be limited to the digital circulation. In fact, microvascular disease is increasingly recognized as a systemic entity, with affected sites comprising the coronary, renal, pulmonary, and cerebral circulations [19]. More precisely, a link between digital microangiopathy and coronary microvascular dysfunction has been described: reduced digital reactive hyperemia, a marker of microvascular endothelial function, has been shown to correlate with coronary microvascular impairment [20, 21 Coronary microvascular disease is itself associated with increased morbidity and mortality and is implicated in heart disease, such as myocardial infarction with non-obstructive coronaries and heart failure with preserved ejection fraction [22]. In a primary prevention setting, reduced hyperemic velocity, a surrogate marker of peripheral endothelial function, was associated with cardiovascular events [23]. Whether the digital microangiopathy observed in patients with incipient RP without previously known connective tissue disease is, however, related to systemic microvascular disease has not been established. In contrast, structural capillary changes beyond functional vasospasm have been acknowledged in a subset of patients with primary RP [24, 25]. Some of these capillary changes are associated with increased ANA-titers, although this association appears to be less pronounced in children [9, 26].

Interestingly, the association between concomitant ANA positivity, pathologic nailfold capillaries, and increased all-cause mortality was observed only in women. Although the male subgroup may have been underpowered to detect a similar association, biological sex differences may also contribute. Women are more likely to exhibit autoimmune phenomena and differ in their immunological responses to self-antigens as well as their innate and adaptive immune responses [27, 28]. Thus, the combination of ANA positivity and microvascular abnormalities may identify a distinct biological phenotype more readily in women. ANA positivity may represent one component of a broader process of systemic immune activation or microvascular dysfunction, with abnormal nailfold capillaries reflecting a manifestation of generalized microangiopathy. Nevertheless, the observed sex-specific association should be interpreted cautiously and requires confirmation in larger cohorts.

Regarding the analysis of ICD-codes obtained from Statistik Austria (Supplementary Fig. 1), the distribution of the causes of death did not differ between patients with both abnormal nailfold capillaries as well as positive autoantibodies and the general study population, with cardiovascular and malignant diseases being the leading causes of death in all groups (Supplementary Fig. 2). Thus, although abnormal nailfold capillaries and positive autoantibodies are related to all-cause mortality, they do not appear to be associated with one particular disease category. The absence of a predominant cause of death suggests that the observed excess mortality is unlikely to be mediated by a single disease process. Rather, the coexistence of ANA positivity and abnormal nailfold capillaries may identify a subgroup with an underlying systemic biological vulnerability that adversely influences outcomes across a broad range of intercurrent illnesses. Whether this reflects generalized microvascular dysfunction, immune dysregulation, or another systemic process remains to be determined.

Finally, the strengths and limitations of this study warrant mention: the lack of a more structured follow-up comprising the potential development of autoimmune rheumatic disease in a subset of study participants as well as key time-varying variables such as prescribed treatments constitute a relevant limitation of this study. Although partially offset by the large number of study participants and the nature of the reference population, itself subjected to a comparable incidence and prevalence of respective comorbid conditions, both study groups cannot be equated in terms of prognosis and the authors cannot rule out confounding bias as a result. Additionally, over an inclusion period of 15 years, different laboratory tests were used for antibody detection, all of which, however, complied with the quality standards for immunologic assay techniques valid at the respective time points. However, sensitivity and specificity of ANA-detection methods deployed throughout the study period may have varied as a result, introducing some heterogeneity with respect to the definition of ANA-positivity in the present study. At the time of this manuscript’s conception, indirect immunofluorescence on Hep-2 cells was still considered the gold standard for the detection of antinuclear antibodies. As testing for ANA subsets is recommended primarily in patients with clinical suspicion of rheumatic spectrum disease, and as the specific immunofluorescence patterns on Hep-2 cells and the commercial availability of the respective diagnostic kits varied over time, ENA subsets were not tested for in all study participants, potentially introducing selection bias. Furthermore, it has to be acknowledged that the mortality analysis stratified by capillaroscopic and serologic abnormalities using Kaplan-Meier Curves does not allow for correction for relevant covariates and should thus be interpreted in the context of the corresponding Cox proportional hazards models. Regarding positive and negative predictive values, it is essential to note that their calculation depends on the prevalence of the respective variables, so the clinical context must be taken into account when interpreting these values. At last, readers of the manuscript should be alerted to the fact that death diagnoses can be inexact and may not always reflect the patient’s leading medical problem.

Regarding the Kaplan-Meier curves, the authors would like to point out that the ‘numbers at risk’ at ten years are deviating from our original manuscript, since the 10-year follow-up was not yet completed for all study participants at the time of its publication and new events for this period have been registered ever since, a fact that is related to the extended inclusion phase of this study. The major strengths of this manuscript are the large number of study participants (*N* = 2922) and the long follow-up duration of 23 years (IQR 22.9–23.1). Additionally, the availability of mortality data from the Austrian reference population obtained from Statistik Austria allows for the comparison of two demographically similar populations, corroborating the results of this analysis. Our results warrant a systematic follow-up of patients with incipient RP presenting with pathognomonic nailfold capillaries aberrations, positive autoantibodies or both, as these findings might be associated with a worse prognosis. Further studies will be necessary to better understand the causes behind this increase in mortality observed in patients with RP without previously known connective tissue disease.

## Supplementary Information

Below is the link to the electronic supplementary material.

Supplementary Material 1

## Data Availability

The data underlying this manuscript will be provided upon reasonable request.

## Declarations

### Disclaimer

No part of this report is copied, published elsewhere, or generated by AI.
