# Supplementary material for: Relation of nailfold capillaries and autoantibodies to mortality in patients with Raynaud’s phenomenon: a retrospective analysis
Source: Rheumatol Int. 2026 Aug 4;46(8):230. doi: 10.1007/s00296-026-06261-7 (PMC13437655; doi:10.1007/s00296-026-06261-7)
Supplement: Supplementary file 1 [file 296_2026_6261_MOESM1_ESM.docx]

**Supplementary Table 1. Associations of Abnormal Nailfold Capillaries and Immunologic Markers as well as Cardiovascular Risk Factors with Mortality in 2922 Patients with Incipient Raynaud Phenomenon (According to Sex)**

|  |  | Females (N = 2268) |  |  | | Males (N = 654) | |  |
| --- | --- | --- | --- | --- | --- | --- | --- | --- |
|  | Hazard Ratio | Confidence Interval | P Value | Hazard Ratio | | Confidence Interval | | P Value |
| Pathognomonic nailfold capillaries | 1.66 | 1.08 – 2.57 | 0.02 | 1.35 | 0.72 – 2.53 | | 0.35 | |
| ANA | 1.52 | 1.00 – 2.30 | 0.05 | 0.61 | 0.21 – 1.75 | | 0.36 | |
| Diabetes mellitus | 0.18 | 0.02 – 1.43 | 0.10 | 3.99 | 2.26 – 7.05 | | <0.001 | |
| Hyperlipidemia | 1.55 | 1.01 – 2.38 | 0.05 | 1.38 | 0.80 – 2.37 | | 0.25 | |
| Smoking | 0.63 | 0.41 – 0.97 | 0.04 | 0.60 | 0.36 – 1.01 | | 0.05 | |
| Obesity (BMI >30) | 1.23 | 1.07 – 1.42 | <0.01 | 0.74 | 0.28 – 1.94 | | 0.54 | |

ANA: antinuclear antibodies, N/A: not applicable due to a low overall prevalence and/or a low number of events in the respective subgroup

**Supplementary Figure 1. Percentage Share of Death Diagnoses Among Deceased Study Participants and Distribution of Groups Among the Main Categories of Death Diagnoses**

Group 1: normal nailfold capillaries, negative autoantibody tests; group2: abnormal nailfold capillaries, negative autoantibody tests; group 3: normal nailfold capillaries, positive autoantibody tests; group 4: abnormal nailfold capillaries, positive autoantibody tests

**Supplementary Figure 2. Percentage Share of Groups Among Deceased Study Participants and Distribution of Death Diagnoses Among the Groups**

Group 1: normal nailfold capillaries, negative autoantibody tests; group2: abnormal nailfold capillaries, negative autoantibody tests; group 3: normal nailfold capillaries, positive autoantibody tests; group 4: abnormal nailfold capillaries, positive autoantibody tests
